# Serogroup B Meningococcal Disease Outbreak and Carriage Evaluation at a College — Rhode Island, 2015

**Published:** 2015-06-12

**Authors:** Heidi M. Soeters, Lucy A. McNamara, Melissa Whaley, Xin Wang, Nicole Alexander-Scott, Koren V. Kanadanian, Catherine M. Kelleher, Jessica MacNeil, Stacey W. Martin, Nathan Raines, Steven Sears, Cynthia Vanner, Jeni Vuong, Utpala Bandy, Kenneth Sicard, Manisha Patel

**Affiliations:** 1 Epidemic Intelligence Service, CDC; 2 Division of Bacterial Diseases, National Center for Immunization and Respiratory Diseases, CDC; 3 Rhode Island Department of Health; 4 Providence College, Providence, Rhode Island; 5 Icahn School of Medicine at Mount Sinai, New York, New York

On February 2, 2015, the Rhode Island Department of Health was notified of a case of meningococcal disease in a male undergraduate student at Providence College. Three days later, a second case was reported in a male undergraduate with no contact with the first student, indicating an attack rate of 44 cases per 100,000 students, nearly 500 times higher than the national incidence of 0.15 cases per 100,000 among persons aged 17–22 years (Division of Bacterial Diseases, National Center for Immunization and Respiratory Diseases, CDC, unpublished data, 2013). Both cases were caused by a rare outbreak strain of *Neisseria meningitidis* serogroup B (ST-9069); neither case was fatal. In response to the outbreak, potential contacts received antibiotic chemoprophylaxis, and a mass vaccination campaign with a recently licensed serogroup B meningococcal (MenB) vaccine was implemented. In collaboration with CDC, the first phase of a meningococcal carriage evaluation was undertaken.

Meningococcal disease is uncommon in the United States but can infect otherwise healthy persons. *N. meningitidis* serogroup B accounts for approximately half of all meningococcal cases among persons aged 17–22 years in the U.S. (Division of Bacterial Diseases, National Center for Immunization and Respiratory Diseases, CDC, unpublished data, 2013) and caused four recent outbreaks in college settings (1,2). *N. meningitidis* is transmitted through direct contact with large-droplet respiratory tract secretions from persons with meningococcal disease or asymptomatic nasopharyngeal carriage (3). Two MenB vaccines, MenB-FHbp (Trumenba, Wyeth Pharmaceuticals, Inc.) and MenB-4C (Bexsero, Novartis Vaccines) were recently licensed in the United States.* Although there are no current recommendations for general use of MenB vaccines, the Advisory Committee on Immunization Practices recommends use of MenB vaccines in persons aged ≥10 years at increased risk for serogroup B meningococcal disease, including in outbreak settings (4). CDC’s interim guidance suggests consideration of vaccination during outbreaks in which two or more primary cases of *N. meningitidis* serogroup B are reported in organizations of <5,000 persons within a 6-month period (5).

As part of the outbreak response, ciprofloxacin chemoprophylaxis (3) was provided to 71 persons who were potentially exposed to oral secretions from either of the two students. Additionally, the school provided education to students on signs and symptoms of meningococcal disease and safe hygiene practices to prevent transmission. Molecular testing on the outbreak strain detected the gene coding for FHbp B24 (6), predicting cross-protection with both MenB vaccines (7).

During 2 vaccination days (February 8 and 11), the first of 3 doses of MenB-FHbp was offered to eligible persons affiliated with Providence College: 1) all undergraduate students; 2) graduate students or staff aged <25 years who lived or worked on campus, 3) persons in an intimate physical relationship with an undergraduate, and 4) asplenic persons or persons with an immunocompromising condition known to place them at risk for meningococcal disease. Persons who declined vaccination were required to sign opt-out forms. Among 3,745 eligible persons, 3,525 (94%) received the first dose. No further college-associated cases were identified as of June 8, 2015.

An evaluation to assess the prevalence of nasopharyngeal carriage of *N. meningitidis* among students and the impact of MenB vaccination on carriage was conducted during February 16–20. Undergraduate students and graduate students who lived on campus were eligible to participate. After obtaining informed consent, an oropharyngeal swab and a short questionnaire assessing risk factors for meningococcal disease and carriage were collected from each participant. Specimens were tested using bacterial culture, real-time polymerase chain reaction, and molecular methods. Log-linear binomial regression models were used to calculate prevalence ratios (PRs) and 95% confidence intervals (CIs).

Of 717 participants in the carriage evaluation, 470 (66%) were female, 655 (91%) lived on campus, and 701 (98%) had received the first MenB-FHbp vaccine dose. Preliminary data indicate that 176 (25%) were carriers of *N. meningitidis*. Among 31 (4%) participants with serogroup B carriage, none carried the outbreak strain. Eight (1%) participants carried serogroup C, one (<1%) carried serogroup X, four (1%) carried serogroup Y, and 132 (18%) carried nongroupable *N. meningitidis*. Males (PR = 1.5, CI = 1.2–2.0), smokers (PR = 1.5, CI = 1.1–2.0), and persons who reported visiting bars or nightclubs or attending parties one or more times per week (PR = 2.7, CI = 1.8–4.2) had increased carriage prevalences, whereas recent antibiotic use was associated with decreased carriage (PR = 0.4, CI = 0.2–0.7).

The baseline carriage prevalence of *N. meningitidis* among Providence College students is comparable to prevalences of up to 34% previously observed among university students in the United Kingdom (8) but is higher than previous U.S. estimates of 1%–8% among the general population (9,10). No carriage of the outbreak strain was detected. There are several possible explanations for this finding. First, the outbreak strain ST-9069 might have a lower propensity for developing a carrier state. Second, the well-targeted chemoprophylaxis strategy, the vaccination campaign, or both, might have eradicated ST-9069 carriage on the campus before the carriage evaluation. Third, our sample size might not have been large enough to detect a very low prevalence of the outbreak strain. A second carriage evaluation was conducted in April; laboratory testing is ongoing, and a third evaluation is planned for the fall of 2015. These additional evaluations will permit assessment of the impact of the MenB vaccination campaign on carriage over time among Providence College students, and might inform recommendations for other college populations.

## Footnotes

* Additional information available at http://www.fda.gov/NewsEvents/Newsroom/PressAnnouncements/ucm431370.htm.
